# Glioma Subtypes Based on the Activity Changes of Immunologic and Hallmark Gene Sets in Cancer

**DOI:** 10.3389/fendo.2022.879233

**Published:** 2022-06-13

**Authors:** Sihan Chen

**Affiliations:** Taikang (Ningbo) Hospital Co., Ltd. Yinzhou, Ningbo, China

**Keywords:** glioma, TCGA, GEO, GSEA, GSVA, cancer, TME

## Abstract

**Purpose:**

Glioma is the most common primary cranial brain tumor that arises from the cancelation of glial cells (which can be in the brain or spinal cord). It is due to innate genetic risk factors or induced by a carcinogenic environment. If left untreated, the disease has a poor prognosis.

**Methods:**

In this study, we downloaded glioma data from TCGA database and GEO (GSE4412). The GSEA database was used to screen tumor microenvironment-related gene sets. Cancer subtypes were classified by GSVA enrichment method.

**Results:**

By GSVA enrichment analysis, we obtain three Gliomas cancer subtypes. After further survival prognosis analysis and biological function analysis, we obtained 13 tumor microenvironment gene sets and 14 core genes that affect patients’ survival prognosis, and these genes have the potential to become targets for targeted therapies and disease detection.

**Conclusion:**

We screened a total of 13 gene sets through a series of enrichment analyses, statistical and prognostic analyses, etc. Among them, 14 core genes were identified, namely: TOP2A, TPX2, BUB1, AURKB, AURKA, CDK1, BUB1B, CCNA2, CCNB2, CDCA8, CDC20, KIF11, KIF20A and KIF2C.

## Introduction

Glioma is the most common primary tumor of the brain, which originates from glial cells. Glial cells nourish and support neuronal cells, but under the induction of some physicochemical factors, neuronal cells can deteriorate into glioma cells (1). Numerous studies have confirmed that the progression of glioma is closely related to the Tumor microenvironment (TME). The tumor microenvironment can induce the differentiation of normal cells into tumor cells through a series of physicochemical mechanisms, so the current study focused on the direct relationship between microenvironmental cells and glioma (1–3). When gliomas are at an early stage, the prognosis for surgery and related treatments is better. However, apart from imaging examinations, there is currently a lack of markers for the early detection of gliomas. And when gliomas progress to an advanced stage, we also need more markers for targeted therapy of gliomas (4). With the establishment of databases such as TCGA and GEO, more and more studies are using big data to analyze therapeutic targets as well as genes available for screening. In the current study, enrichment analysis was used to screen tumor microenvironment-related genes and classify glioma into three subtypes, and finally screen the gene set and core genes that affect the survival prognosis of glioma patients (5). In conclusion, we mined 21 sets of microenvironmental gene sets and 17 core genes affecting glioma survival prognosis through a series of statistical analysis and enrichment analysis (6).

## Methods

### Data Collection

For the present study, the included genetic and clinical data were downloaded from the TCGA database. We collected 508 cases of gene expression data, and 501 cases of clinical data from glioma patients. In addition, we downloaded 4922 immune gene datasets from Gene Set Enrichment Analysis (GSEA) (https://www.gsea-msigdb.org/gsea/msigdb/index.jsp) (7).

### Functional Enrichment Analysis Using GSVA

Gene set variation analysis (GSVA) can be used to assess the degree of enrichment of a specific gene set in a sample population and thus observe changes in the activity of a set of gene sets. Enrichment analysis of the above gene set was performed using GSVA to assess the relevant biological activity (8).

### Statistical Analysis

All statistical analyses were performed with R software (version 4.1.2). The normally distributed measurement data were compared between groups using the student’s t test, and the non-normally distributed measurement data were compared using the Wilcoxon Signed Rank Test. Kaplan-Meier (K-M) survival curves were used to describe the differences in survival between the two groups. heatmap and subtype classification of glioma samples were analyzed by the Heatmap and CancerSubtys packages of the R software. Apply NMF package to cancer genomic dataset for NMF. least absolute shrinkage and selection operator (LASSO) were used to screen for sets of genes that potentially affect patient survival prognosis. p-values <0.05 were considered statistically significant. p<0.01 was considered a significant difference. Risk score calculation formula: expression mRNA1 × coefficient mRNA1+ expression rmRNA2 × coefficient fmRNA2 +…+ expression mRNAn ×coefficient mRNAn (**Table 1**). The AUC (Area Under roc Curve) value of ROC indicates that 0.5-0.7 is acceptable, 0.7-0.9 is good, and> 0.9 is excellent.

**Table 1:** Results of LASSO analysis regarding 13 gene sets affecting the survival prognosis of glioma patients.

Gene set	Coef
T_GSE34205_HEALTHY_VS_FLU_INF_INFANT_PBMC_DN	8.757127
T_GSE27241_WT_VS_RORGT_KO_TH17_POLARIZED_CD4_TCELL_UP	4.576126
T_GSE32901_NAIVE_VS_TH1_CD4_TCELL_DN	-8.93348
T_GSE24634_TREG_VS_TCONV_POST_DAY3_IL4_CONVERSION_UP	-3.68063
T_GSE5589_LPS_AND_IL10_VS_LPS_AND_IL6_STIM_MACROPHAGE_45MIN_DN	-0.96574
T_GSE22886_UNSTIM_VS_IL2_STIM_NKCELL_DN	-4.45902
T_HALLMARK_ANGIOGENESIS	-1.0114
T_GSE19941_LPS_VS_LPS_AND_IL10_STIM_IL10_KO_MACROPHAGE_UP	14.59616
T_GSE29614_CTRL_VS_TIV_FLU_VACCINE_PBMC_2007_DN	-9.42
T_GSE13547_2H_VS_12_H_ANTI_IGM_STIM_BCELL_DN	-3.26356
T_GSE45365_WT_VS_IFNAR_KO_BCELL_DN	-0.15757
T_GSE14415_ACT_TCONV_VS_ACT_NATURAL_TREG_DN	20.46846
T_GSE22313_HEALTHY_VS_SLE_MOUSE_CD4_TCELL_DN	-4.85501

### Protein Interaction Networks and Biological Function Networks

STRING (https://cn.string-db.org/) was used to analyze the interaction links between genes, and then the core genes of the protein interaction network were obtained using the plug-in MCODE of Cytoscape software (version 3.9.0). the Clue GO plug-in was used to generate the biological functional interaction network (9).

## Results

### Immunological Activity of the Hallmark Gene Set in the Glioma Samples

Because GSVA can detect even subtle pathway activity, we use GSVA to detect pathway activity in selected sets of genes. We downloaded 4922 immunologically relevant genes from GSEA to provide a comprehensive picture of the changes in the immune activity of glioma. gene expression of glioma was downloaded from the TCGA database (from 508 glioma patients) and GEO (GSE4412, 85 glioma patients).

**Figure 1:**
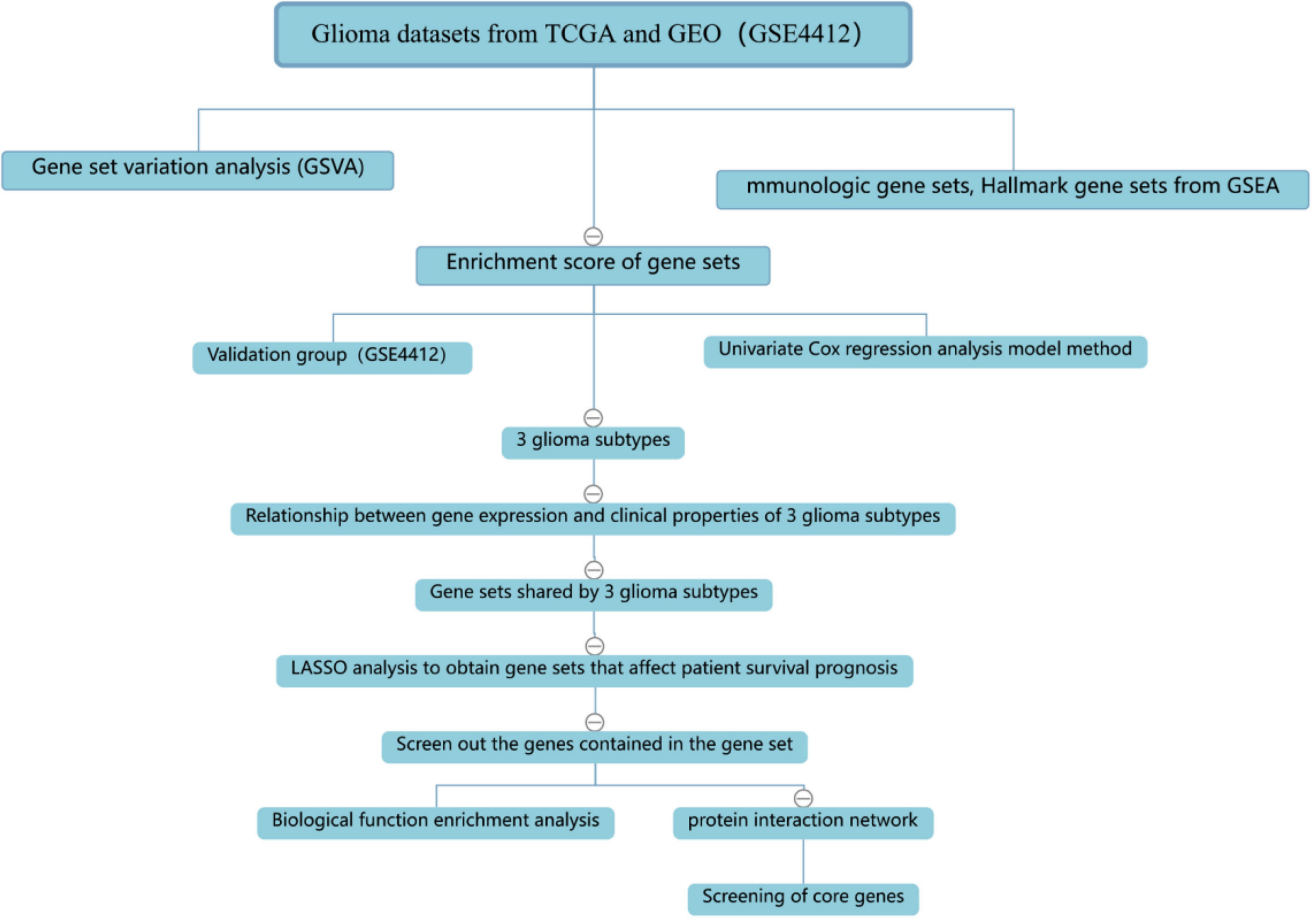
Flowchart about our research.

**Figure 2:**
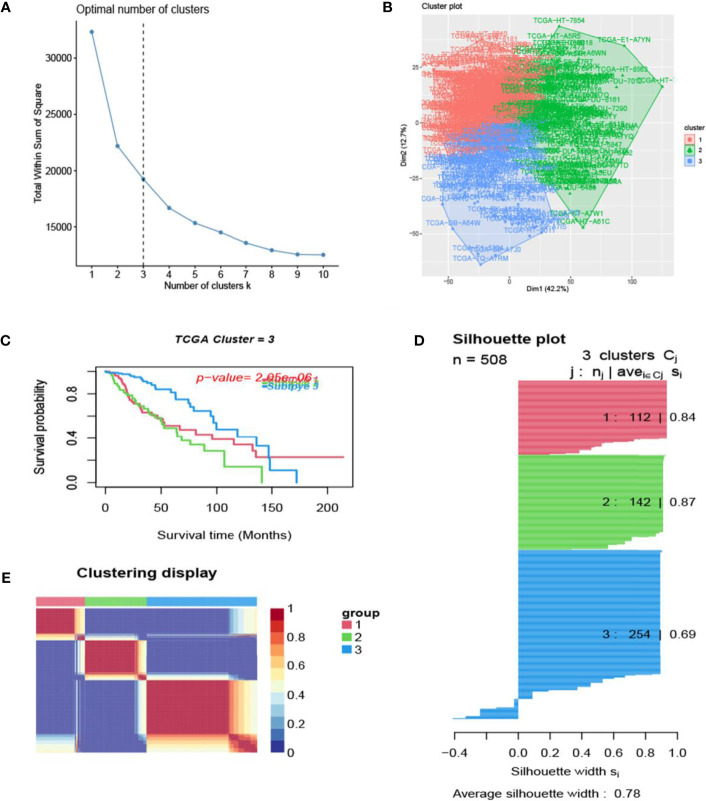
Glioma subtype identification (TCGA). **(A)** Use the factoextra package to perform clusters analysis. **(B)** Sample composition diagram of the 3 cancer subtypes. **(C)** K-M curves of different cancer subtypes. **(D)** Cancer Subtypes’ Silhouette width plots. **(E)** The clustering displays of the three cancer subtypes.

**Figure 3:**
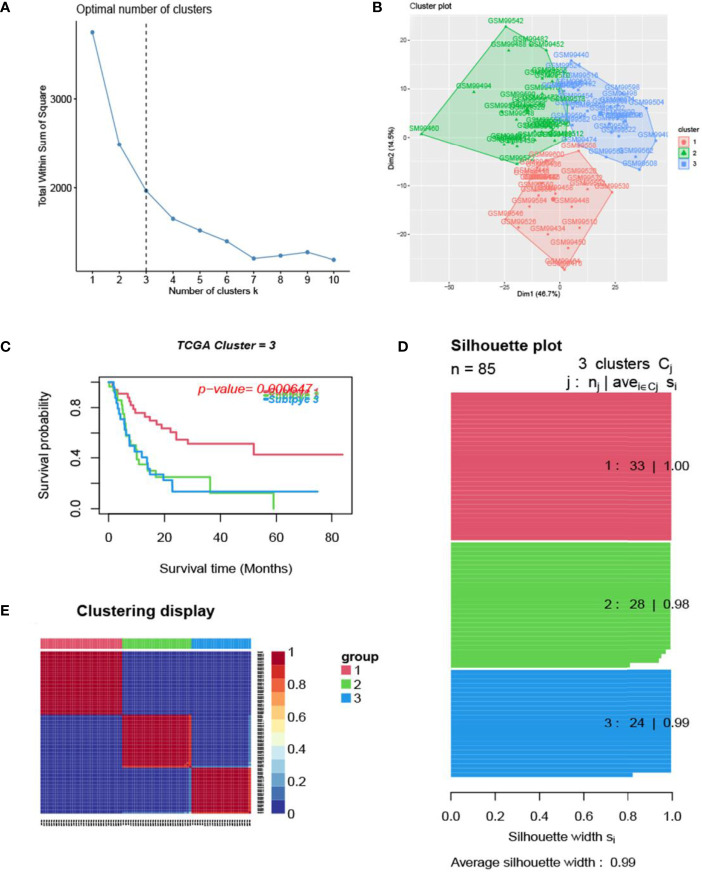
Glioma subtype identification (GEO GSE4412). **(A)** Use the factoextra package to perform clusters analysis. **(B)** Sample composition diagram of the 3 cancer subtypes. **(C)** K-M curves of different cancer subtypes. **(D)** Cancer Subtypes’ Silhouette width plots. **(E)** The clustering displays of the three cancer subtypes.

The process of the whole study is placed in **Figure 1**. After obtaining gene expression amounts, we tried to classify glioma patients into different subtypes. Because survival prognosis data were available for only 508 patients in TCGA, after collation, we screened the data from 508 patients who had both gene expression data and clinically relevant data. Using the LASSO (Least absolute shrinkage and selection operator), Nonnegative matrix factorization and CancerSubtypes package, we classified glioma into 3 subtypes (**Figure 2E**). The average Silhouette width value was 0.88 (the closer the value is to 1 the more accurate it is, and 0.88 is already a very accurate value) (**Figure 2D**). The factoextra package of R software can classify cases into the best groups (**Figures 2A, B**). Kaplan-Meierplotter analysis was used to construct a survival model. Among the three subtypes of glioma, subtype 1 had the worst prognosis, subtype 2 was slightly better, while subtype 3 had the best prognosis among the three subtypes (**Figure 2C**). The data in GEO (GSE4412) was analyzed using the same method described above and the corresponding results are placed in **Figure 3**.

### Relationship Between Glioma Tumor Subtypes and Clinical Information

**Figure 4:**
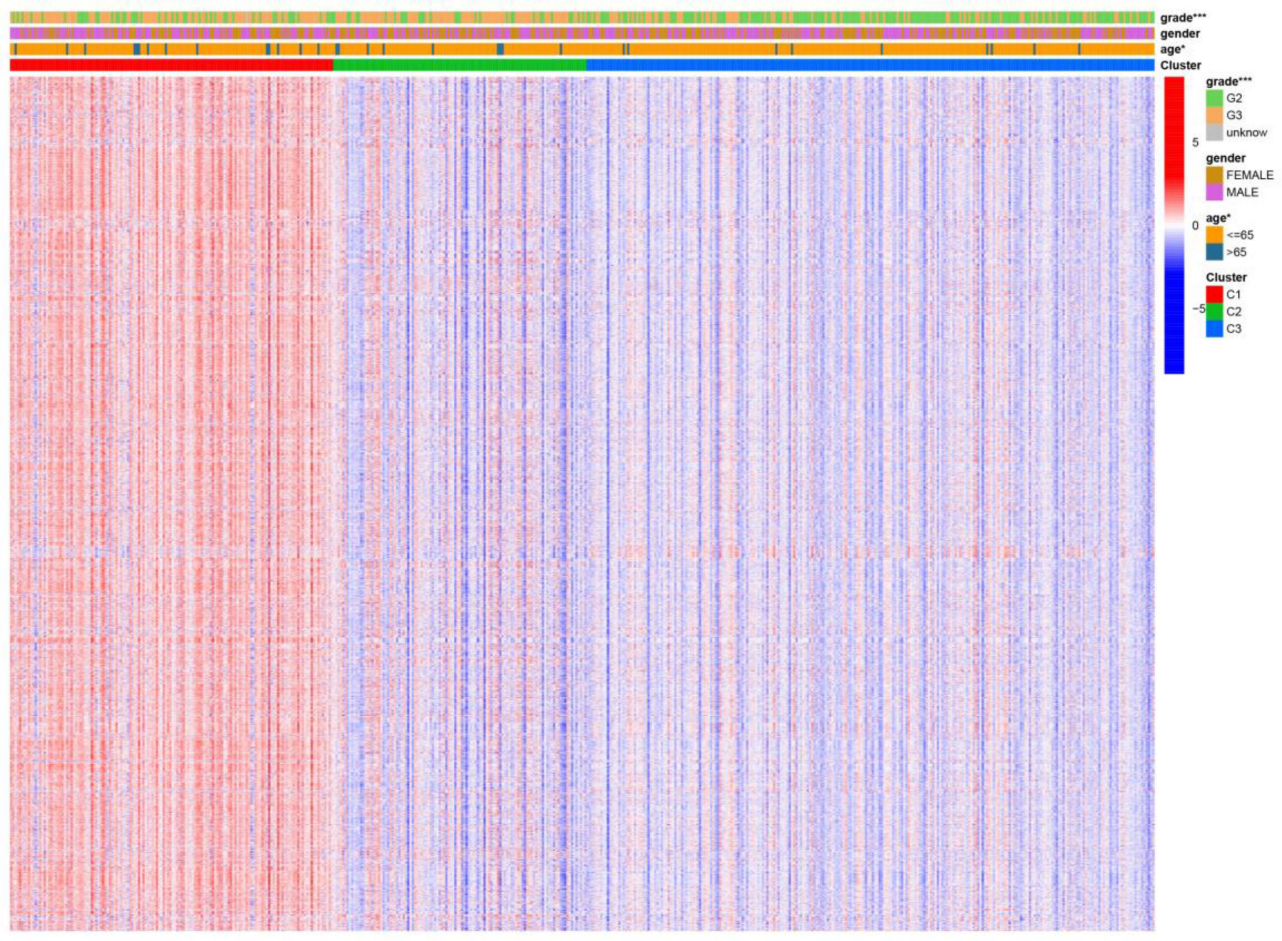
Differential gene sets’ expression of clinical features and cancer subtypes. * means P<0.05, and *** means P<0.001.

**Figure 5:**
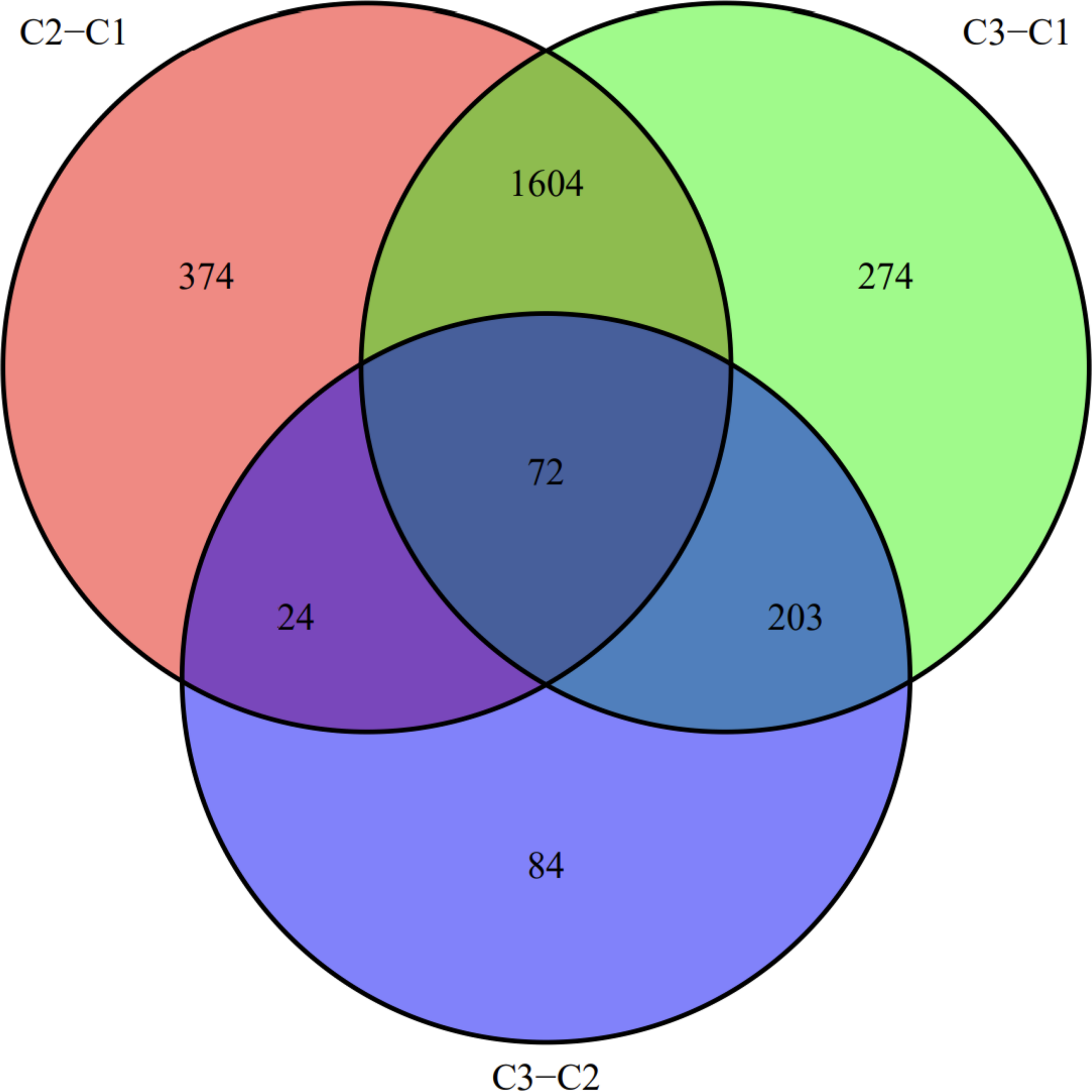
Venn diagram, a common set of differential genes for the three cancer subtypes.

**Figure 6:**
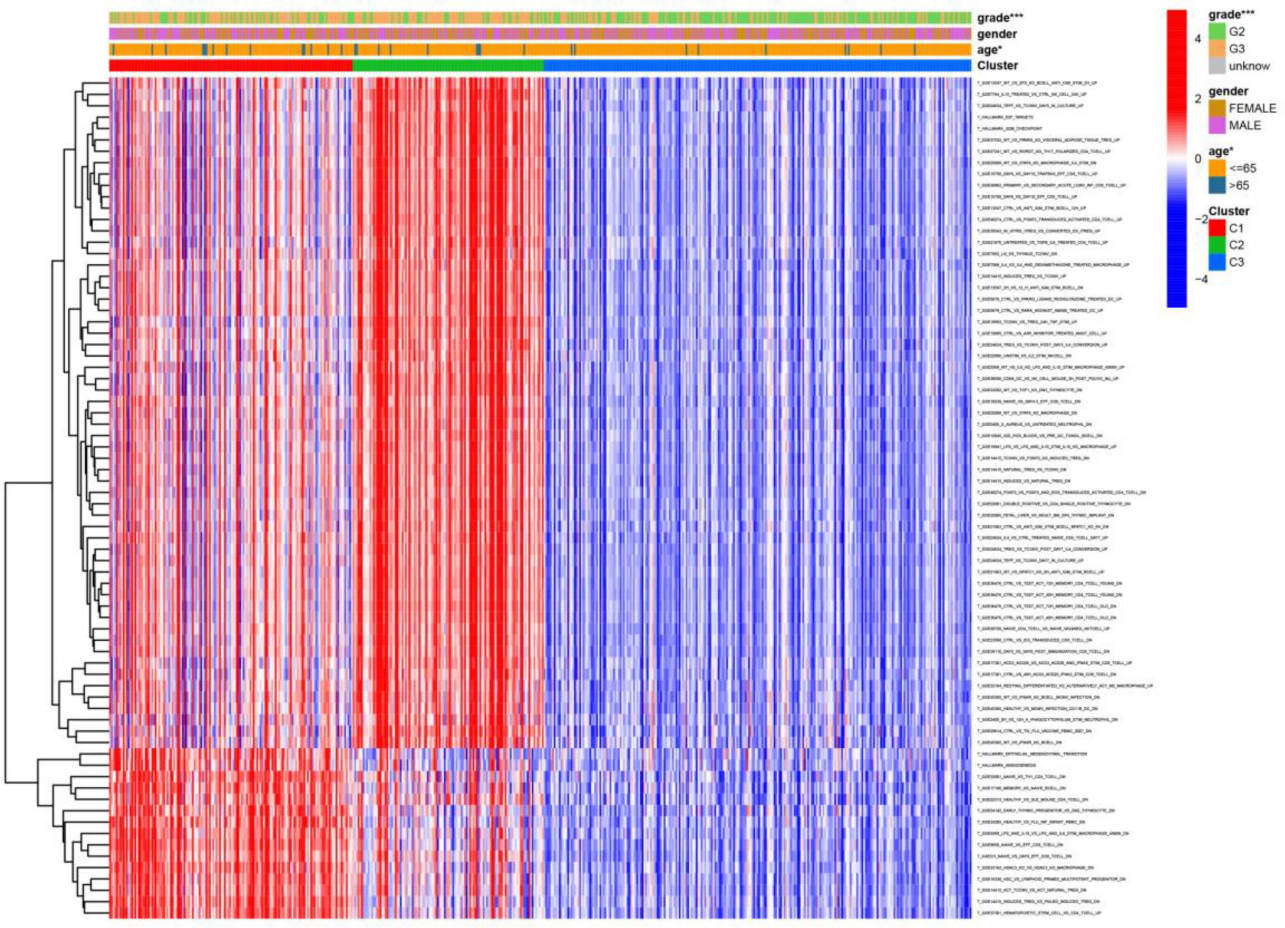
Heat map of the relationship between the expression of gene sets affecting patient survival prognosis, and individual clinically relevant information. * means P<0.05, and *** means P<0.001.

We visualized the relationship between clinical information and glioma gene expression by heat map. The results showed that cancer subtypes were strongly correlated with glioma grade, P < 0.001, and cancer subtypes were correlated with patient age, P < 0.05. The above two clinical information were statistically significant between different cancer subtypes. The specific information is shown in **Figure 4**. Visualization of the differential gene sets of the three cancer subtypes by Venn diagram showed that there were 72 differential gene sets among the three cancer subtypes (**Figure 5**). Visual analysis of these 72 gene sets showed significant differences in the expression of the 72 gene sets in the three different cancer subtypes (**Figure 6**).

### Linking Gene Expression and Survival Prognosis of Glioma Patients

**Figure 7:**
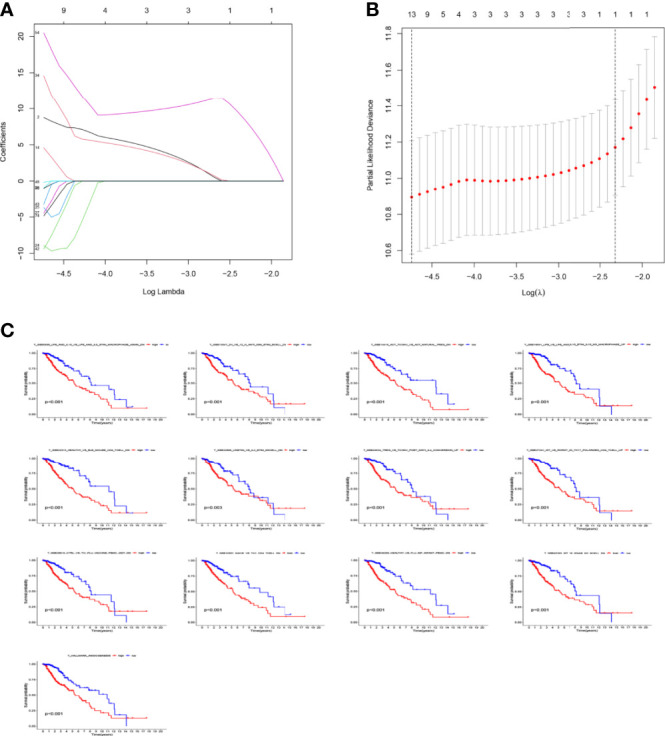
Prognostic gene sets in glioma. **(A)** LASSO coefficient profiles (y-axis) of the gene sets and the optimal penalization coefficient (λ) *via* 3-fold cross-validation based on partial likelihood deviance. **(B)** The dotted vertical lines represent the optimal values of λ. The top x-axis has the numbers of gene sets, whereas the lower x-axis revealed the log (λ). **(C)** High enrichment scores in glioma were associated with poor overall survival. P-values <0.05 were considered statistically significant.

**Figure 8:**
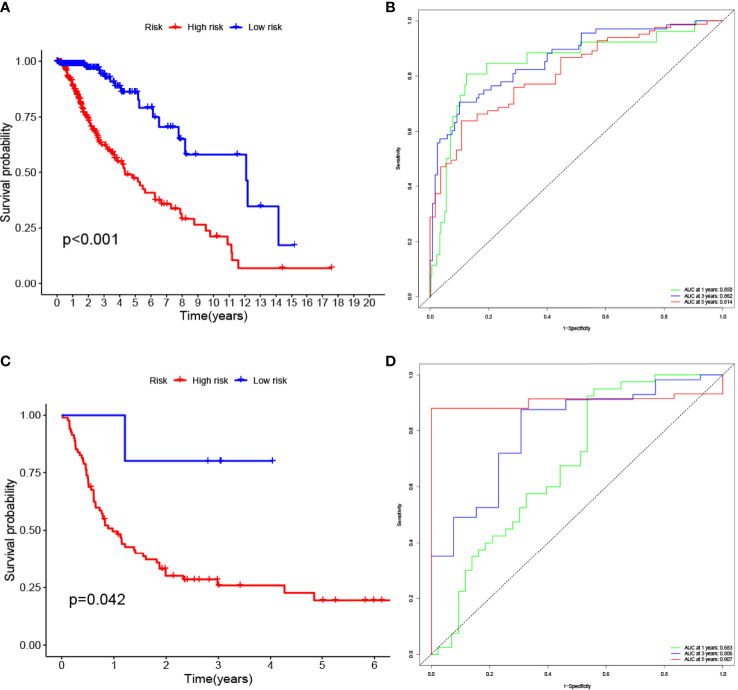
High risk score among glioma patients were associated with low overall survival (p-value < 0.05 was considered statistically significant). The AUC (Area Under roc Curve) value of ROC indicates that 0.5-0.7 is acceptable, 0.7-0.9 is good, and> 0.9 is excellent. **(A)** K-M curves of the glioma (TCGA) risk score model. **(B)** glioma (TCGA) ROC curve. **(C)** K-M curves of the glioma (GEO GSE4412) risk score model. **(D)** glioma (GEO GSE4412) ROC curve.

The LASSO allows a good assessment of the relationship between variables on survival and posterior. By LASSO analysis, we concluded that a total of 13 gene sets were associated with the survival prognosis of glioma patients (**Figures 7A, B**). The K-M curves of the 13 gene sets (They affect patient survival prognosis) are shown in **Figure 7C**. The risk scores derived from LASSO were divided into high and low risk based on median, with the high-risk group having a significantly worse survival prognosis than the low-risk group (**Figures 8A, C**). The ROC curve of its survival prognosis is shown in **Figures 8B, D**.

### Screening and Biological Function Analysis of Core Genes

**Figure 9:**
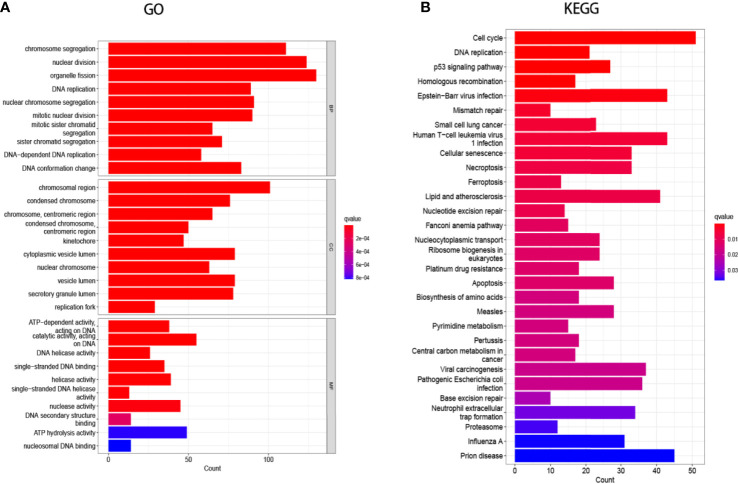
Gene Ontology (GO) and Kyoto Encyclopedia of Genes and Genomes (KEGG) enrichment analysis. **(A)** GO analysis of the 13-group gene set. **(B)** KEGG analysis of the 13-group gene set.

**Figure 10:**
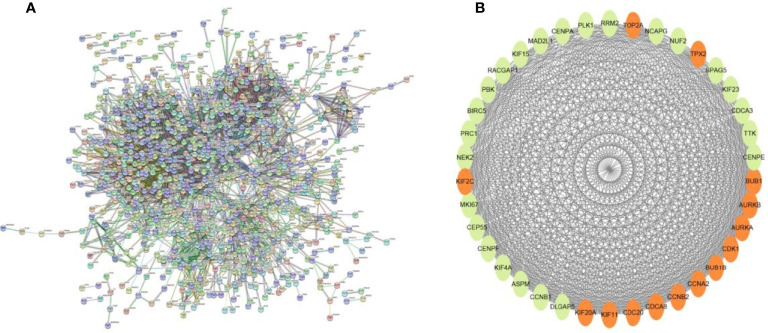
**(A)** PPI network diagram of 13 sets of genes. **(B)** The 14 core gene networks are related to 13 gene sets, namely: TOP2A, TPX2, BUB1, AURKB, AURKA, CDK1, BUB1B, CCNA2, CCNB2, CDCA8, CDC20, KIF11, KIF20A and KIF2C.

Searching the above 13 sets of gene sets in GSEA’s, we obtained 4107 immunology-related genes. The protein interaction network was constructed by STRING database (https://cn.string-db.org/). The DAVID database was used to analyze the GO and KEGG functions of the 13 gene sets obtained above (**Figure 9**). The genes in the 13 gene sets were selected to make a protein interaction network, and Cytoscape software was used to screen out the core modules in the PPI network (**Figure 10A**). Finally, the core modules and core genes in the protein interaction network were identified using the MCODE plugin. The results showed that a total of 14 core genes were filtered out. They are TOP2A, TPX2, BUB1, AURKB, AURKA, CDK1, BUB1B, CCNA2, CCNB2, CDCA8, CDC20, KIF11, KIF20A and KIF2C (**Figure 10B**).

## Discussion

Glioma is a common primary tumor of the central nervous system. The microenvironment of the glioma tissue area consists of tumor cells, immune cells and various factors secreted by them. Among them, various factors secreted by tumor cells or immune cells, such as growth factors, chemokines, pro-inflammatory factors and anti-inflammatory factors, form the microenvironmental network, which interact with each other to regulate and influence the process of tumor (10).

Glial cells support nerve cells in their associated neural activities and keep them healthy. However, glial cells can also give rise to a malignant tumor, glioma, which is the most common primary malignant tumor of the brain. As glioma invasion progresses, its prognosis gradually deteriorates. At present, in addition to a few detection methods such as imaging, there is a lack of corresponding markers to detect the occurrence and progression of gliomas, and there is also a lack of targets for chemotherapy in the later stages of gliomas. The differences in symptoms are mainly related to the location where the glioma occurs. In addition to causing many painful symptoms, gliomas can also greatly reduce the survival time of patients. However, there is still a great deal of research into the treatment and detection of gliomas in the brain. With the establishment of databases like TCGA, GEO and GSEA, more and more studies are using big data and AI to analyze many valuable research results. Many studies have now found that immunologically relevant activities of tumors, play an important role in both suppressing and promoting tumor progression. The present study will focus on immunology-related activities to analyze glioma-related mechanisms and clinical prognosis. In this study, we used a database with each analysis software to analyze the big data of glioma patients, aiming at obtaining some gene sets that can predict the prognosis of patients’ survival. These gene sets are expected to be new targets for detection or treatment.

In this study, we screened the gene expression transcripts of glioma in TCGA database and downloaded their corresponding clinical data. We downloaded gene expression data for a total of 508 patients, but their corresponding clinical data were available for only 501cases. Therefore, we deleted 7 cases of gene expression data without clinical data. We downloaded immune-related gene sets from GSEA and then enriched 3 different cancer subtypes by GSVA. Among the three different cancer subtypes, subtype 1 has the worst survival prognosis, subtype 3 has the best prognosis, and subtype 2 is in between. The closer the Silhouette width value is to 1, the higher the accuracy of the model is represented. Among the three different cancer subtypes, the highest Silhouette width value was 0.84 for subtype 1, 0.87 for subtype 2, and 0.69 for subtype 3. The average Silhouette width value of the three subtypes was 0.78, which represents the high accuracy of our model. With the nonnegative matrix factorization (NMF) method, we can clearly see that the 3 cancer subtypes have different Clustering displays (**Figure 2**). This also represents that there is a significant difference in gene expression among the three cancer subtypes. The same method was used to detect information about 85 patients in GEO (GSE4412). The results showed that the three tumor subtypes were 1.00, 0.98 and 0.99 Silhouette plot (Average silhouette width: 0.99) (**Figure 3**). The heat map shows that there are significant differences in gene expression profiles among the three cancer subtypes (**Figure 4**). According to the analysis, the main reasons for this difference were the age of the patients and the grade of the cancer. Using the Venn diagram, we can find 72 common differential gene sets for the 3 cancer subtypes (**Figure 5**). Also, the heat map can show that the three cancer subtypes have significantly different expressions in these 72 gene sets (**Figure 6**). By LASSO regression analysis, we obtained 13 gene sets that affect the prognosis of glioma patients (**Table 1**). K-M curves allow analysis of the relationship between gene set expression and patient survival prognosis. By the median of gene set expression, we divided them into high-risk and low-risk groups, and the results showed that in the 13 gene sets, the survival prognosis of the high-risk group was significantly worse than that of the low-risk group (**Figure 7**). The gene set expression was multiplied by the Coef value of the gene set by LASSO analysis, relying on the median to classify them into high-risk and low-risk groups. K-M curve analysis showed that the survival prognosis of the high-risk group was significantly worse than that of the low-risk group (**Figure 8**). It is worth mentioning that the ROC’s AUC values of the survival models are all greater than 0.6, indicating that our prognostic model has good accuracy. We listed the gene data from 13 gene sets to construct their biological function enrichment. The results show that the core functions of the network are: GO analysis mainly focused on extracellular matrix organization, extracellular structure organization and external encapsulating structure organization. KEGG mainly focused on, Focal adhesion, Proteoglycans in cancer and PI3K-Akt signaling pathway (**Figure 9**). The 13 gene sets were placed into the STRING database and their protein interaction networks were analyzed. The core modules in the protein interaction network were then analyzed by the Cytoscape (3.9.0) plugin MCODE. Finally, we analyzed 14 core genes, which are: TOP2A, TPX2, BUB1, AURKB, AURKA, CDK1, BUB1B, CCNA2, CCNB2, CDCA8, CDC20, KIF11, KIF20A and KIF2C (**Figure 10**). TOP2A is a major target for many targeted therapies, and it is overexpressed in many tumors. It has been shown that inhibition of TOP2A expression inhibits the proliferation of tumor cells in cellular experiments. However, the research on this gene has been limited to basic experiments and has not been well applied to clinical studies (11, 12).. TPX2 can stimulate the proliferation, invasion and metastasis of tumor cells through the AKT pathway (13). AURKB may help gliomas of the brain become resistant to chemotherapy (14). CCNB2 is a member of the cell cycle protein family, specifically the B-type cell cycle proteins. Current studies support that glioma patients with high CCNB2 expression have a significantly worse survival prognosis than those with low CCNB2 expression. However, the exact mechanism is not clear. It is simply described to be closely related to the tumor microenvironment (15–18). AURKA is used as a target in many tumors for targeted therapy, and these genes are also microenvironment constituent genes of tumors (19, 20). CKD1 has not been studied much in glioma. It has been reported that gliomas can develop resistance to Temozolomide by immune escape through the CDK1/survivin signaling pathway (21). BUB1B may provide a marker to predict aggressive and effective drug response (22). CCNA2 has been little studied in glioma, and its mechanism of action in glioma needs to be further investigated (18). CDCA8 acts as a cell cycle regulator and tumor promoter in gliomas and promotes tumor cell proliferation (23). CDC20 overexpression and its associated gene modules were characteristically elevated, signifying increased genomic instability in gliomas (24). KIF11 is a driver of glioma invasion, proliferation and self-renewal (25). A related study showed that tumor specimens from patients with glioma were used for gene sequencing and showed that patients with increased KIF20A expression had a poorer survival prognosis. However, the study of this gene in glioma remains to be further explored (26–28). At present, there are few studies of other core genes in glioma to conclude the mechanisms involved.

In summary, we screened a total of 13 gene sets through a series of enrichment analyses, statistical and prognostic analyses, etc. Among them, 14 core genes were identified, namely: TOP2A, TPX2, BUB1, AURKB, AURKA, CDK1, BUB1B, CCNA2, CCNB2, CDCA8, CDC20, KIF11, KIF20A and KIF2C.

## Data Availability Statement

The datasets presented in this study can be found in online repositories. The names of the repository/repositories and accession number(s) can be found in the article/supplementary material.

## Author Contributions

SC guided and revised the manuscript. The author confirms being the sole contributor of this work and has approved it for publication.

## Funding

This paper is financially supported by Taikang (Ningbo) Hospital Co., Ltd. The author declares that the funder was not involved in the study design, collection, analysis, interpretation of data, the writing of this article or the decision to submit it for publication.

## Conflict of Interest

Sihan Chen was employed by the company: Taikang (Ningbo) Hospital Co., Ltd.

## Publisher’s Note

All claims expressed in this article are solely those of the authors and do not necessarily represent those of their affiliated organizations, or those of the publisher, the editors and the reviewers. Any product that may be evaluated in this article, or claim that may be made by its manufacturer, is not guaranteed or endorsed by the publisher.

## Abbreviations

LASSO, Least absolute shrinkage and selection operator; GSVA, Gene set variation analysis; LGG, low-grade gliomas; WHO, World Health Organization; TME, tumor microenvironment; OS, overall survival; GSEA, gene set enrichment analysis; HR:hazard ratio; TCGA, The Cancer Genome Atlas. GO, Gene Ontology. KEGG, Kyoto Encyclopedia of Genes and Genomes.
